# Noncirrhotic Portal Hypertension due to Nodular Regenerative Hyperplasia Treated with Surgical Portacaval Shunt

**DOI:** 10.1155/2012/965304

**Published:** 2012-08-21

**Authors:** Lisa M. Louwers, Jared Bortman, Alan Koffron, Veslav Stecevic, Steven Cohn, Vandad Raofi

**Affiliations:** ^1^Department of General Surgery, William Beaumont Hospital, Beaumont Health System, Royal Oak, MI 48073, USA; ^2^Department of Gastroenterology, William Beaumont Hospital, Beaumont Health System, Royal Oak, MI 48073, USA; ^3^Division of Hepatobiliary and Transplant Surgery, Department of General Surgery, William Beaumont Hospital, Beaumont Health System, Royal Oak, MI 48073, USA

## Abstract

Nodular regenerative hyperplasia (NRH) is an uncommon condition, but an important cause of noncirrhotic intrahepatic portal hypertension (NCIPH), characterized by micronodules of regenerative hepatocytes throughout the liver without intervening fibrous septae. Herein, we present a case of a thirty-seven-year-old female with systemic lupus erythematosus (SLE) who was discovered to have significant esophageal varices on endoscopy for dyspepsia. Her labs revealed a slight elevation in the alkaline phosphatase and mild thrombocytopenia. Abdominal MRI revealed seven focal hepatic masses, splenomegaly, no ascites, and a patent portal vein. Ultrasound-guided core biopsy was reported as focal nodular hyperplasia. However, her varices persisted despite treatment with beta-blockers and four additional upper endoscopies with banding. She was subsequently referred for a surgical opinion. At that time, given her history of SLE, azathioprine use, and portal hypertension, suspicion for NRH was raised. Given her normal synthetic function and lack of parenchymal liver disease, the patient was offered surgical shunting. During shunt surgery, a liver wedge biopsy was also performed and this confirmed NRH. An upper endoscopy six weeks after shunting verified complete resolution of varices. Currently, fifteen months after surgery duplex ultrasonography demonstrates shunt patency and the patient is without recurrence of her portal hypertension.

## 1. Introduction

Nodular regenerative hyperplasia (NRH) is a rare cause of noncirrhotic portal hypertension, with an incidence in autopsy studies of 0.5–2.6% [1, 2]. The first case of NRH was described by Ranstrom in 1953 in a patient with Felty's syndrome [3], but it was Steiner who coined the phrase “nodular regenerative hyperplasia” in 1959 [4]. Symptomatic patients present with evidence of increased portal venous pressure in the absence of documented parenchymal liver disease or portal vein thrombosis. NRH is usually associated with collagen vascular diseases, azathioprine (AZA) use, antiretroviral therapy in human immunodeficiency virus (HIV) treatment, and postchemotherapy especially with the use of oxaliplatin [5–10].

This paper describes a case report of NRH that presented with advanced esophageal varices and portal gastropathy that did not respond to medical management. The patient underwent nonselective portacaval shunting with resolution of her esophageal varices by six weeks postoperatively.

## 2. Case Report

**Figure 1:**
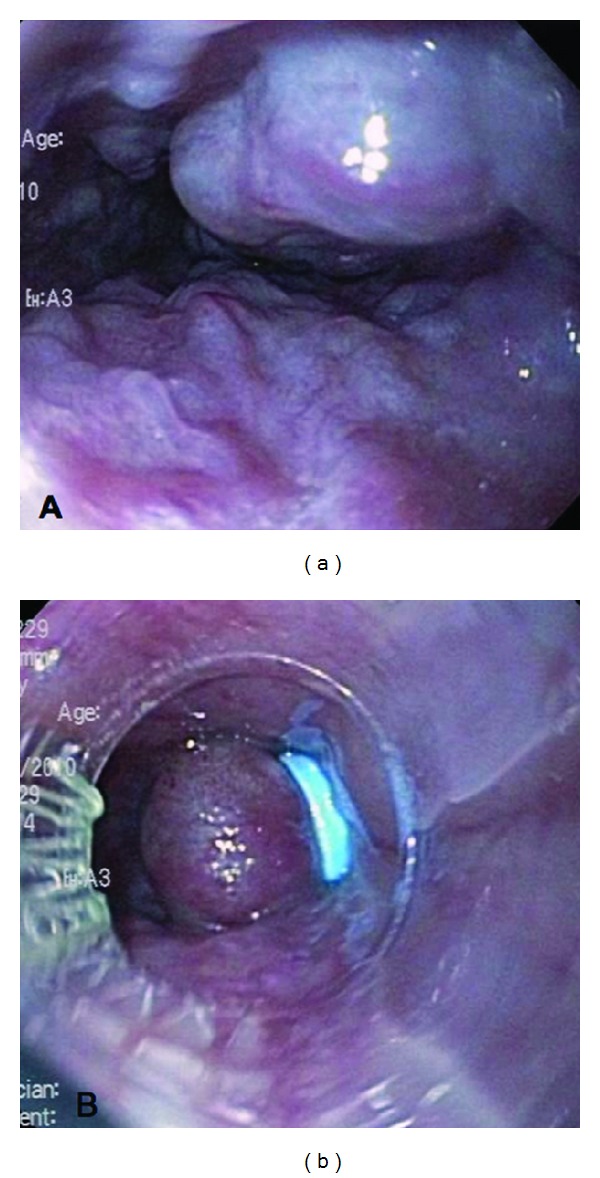
(a) Grade III esophageal varices on index esophagogastroduodenoscopy. (b) Status after endoscopic banding of esophageal varices.

**Figure 2:**
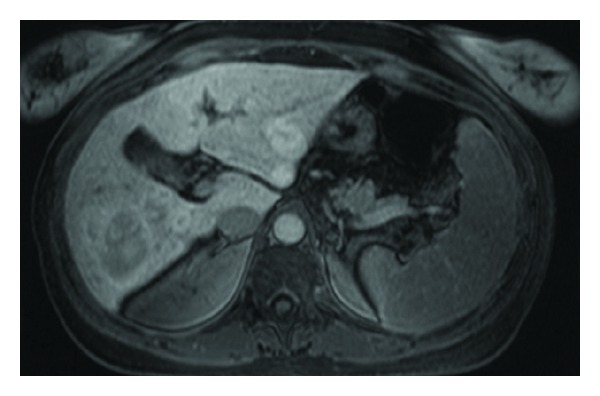
MRI abdomen with and without IV contrast. Multiple masses were scattered throughout the liver. The largest mass in right hepatic lobe measured 4.5 cm.

**Figure 3:**
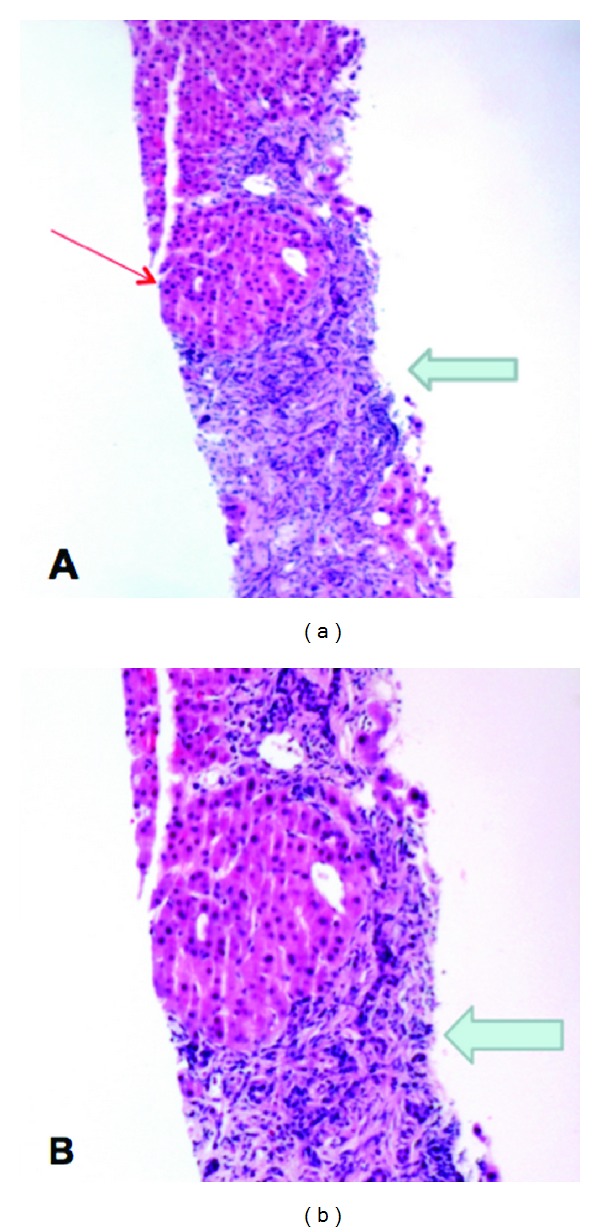
Needle core biopsy of liver mass. (a) Medium power view (5x magnification) demonstrating lobules of hepatic parenchyma (red arrow) separated by broad bands of fibrous connective tissue (blue arrow). (b) Higher power view (10x magnification) demonstrating bile ductular proliferation in the broad fibrous septae.

A 37-year-old woman with systemic lupus erythematosus (SLE) was discovered to have incidental grade III esophageal varices and significant gastropathy during an endoscopy for dyspepsia (Figure 1(a)). Laboratory evaluation was normal except for a mildly elevated alkaline phosphatase of 135 U/L (range 30–110) and a platelet count of 137,000 mil/L (range 150,000–400,000). The patient was otherwise asymptomatic, without any other sequelae of advanced liver disease including jaundice, ascites, or encephalopathy. Further workup with colonoscopy demonstrated diffuse vascular congestion consistent with portal hypertensive colopathy. Abdominal magnetic resonance imaging (MRI) revealed seven focal hepatic masses, a patent portal vein, and a recanalized umbilical vein (Figure 2). An ultrasound-guided core needle biopsy of the largest liver lesion was reported as most consistent with focal nodular hyperplasia (FNH), without surrounding cirrhosis (Figure 3). Over the next 16 months, the patient was treated with nonselective betablockers and four additional upper endoscopies with banding (Figure 1(b)). Despite this, she had persistent and recurrent large varices.

**Figure 4:**
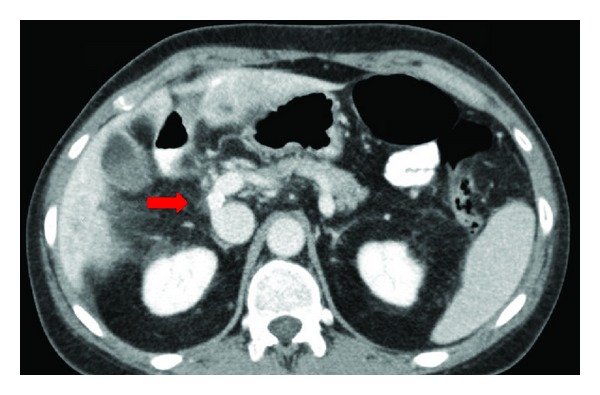
CT abdomen with IV contrast two weeks following surgical portacaval shunt placement. An 8 mm polytetrafluorethylene (PTFE) graft connects the main portal vein with infrahepatic inferior vena cava (red arrow).

**Figure 5:**
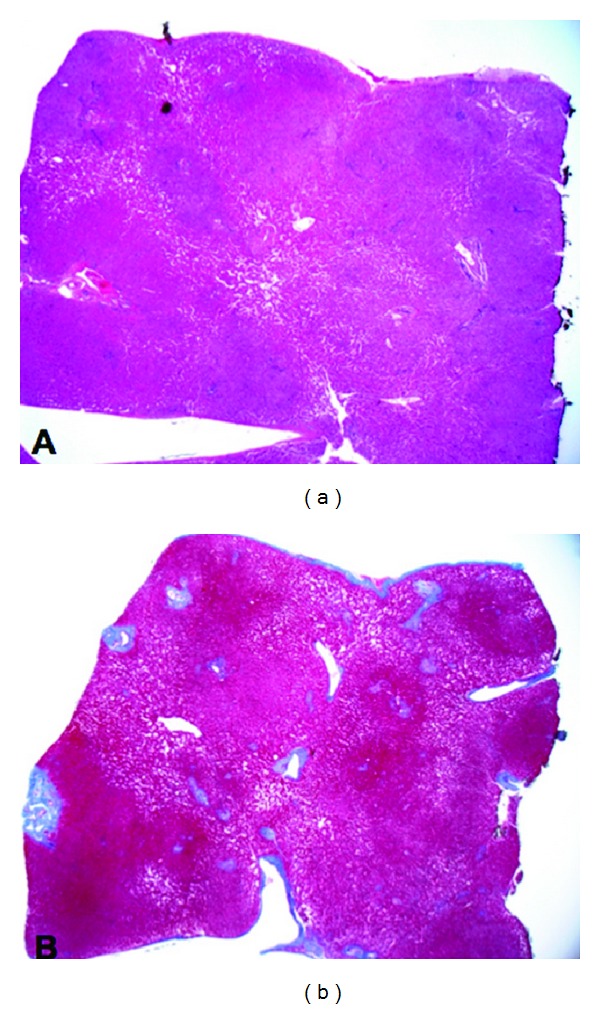
Liver wedge resection. (a) This is a wedge resection specimen demonstrating multiple vaguely defined nodules which are hypercellular. These nodules are diffusely presented and are centered around portal triads. There is no steatosis, cholestasis, or lobular inflammation. (b) The trichrome stain accentuates the nodular pattern and reveals no significant fibrosis. These morphologic features are diagnostic of nodular regenerative hyperplasia.

Given these findings, she was eventually referred for surgical evaluation. Even though her biopsy was reported as FNH, given the patient's history of SLE, AZA use, and signs of portal hypertension, suspicion for NRH was raised. Initially, after consulting with her rheumatologist, the patient stopped taking AZA. However, there was no improvement in her portal hypertension, and given the refractory nature of the patient's varices we elected to pursue more definitive management. The patient therefore subsequently underwent a side-to-side portacaval shunt with an 8 mm polytetrafluorethylene (PTFE) graft (Figure 4). Intraoperatively, the portal pressure prior to shunting was measured at 20 mmHg (normal < 6 mmHg), which subsequently decreased to 5 mmHg after shunt placement. A wedge liver biopsy was also performed at the time of surgery. The final pathology demonstrated multiple hypercellular nodules centered on portal triads without fibrosis, confirming NRH (Figure 5). Postoperatively, the patient did well, with an endoscopy 6 weeks after shunting demonstrating complete resolution of esophageal varices with only mild residual gastropathy. Five months postoperatively, the patient had no deterioration in her liver function tests and no evidence of encephalopathy. Her dyspepsia has also resolved. A duplex ultrasound performed fifteen months postoperatively confirmed patency of the shunt.

## 3. Discussion

It is hypothesized that NRH is not a distinct clinical entity, but rather a disease along a spectrum of microvascular disorders including hepatoportal sclerosis, incomplete septal cirrhosis, and sinusoidal obstruction syndrome. Grossly, the liver has diffuse fine nodularity with 1–3 mm nodules and can resemble micronodular cirrhosis on initial evaluation. These lesions can coalesce into larger tumors and appear pale compared to surrounding hepatic parenchyma. Microscopically, biopsy specimens of NRH contain two types of cells: central-hypertrophied hepatocytes surrounded peripherally by atrophic hepatocytes [11].

The characteristic appearance of NRH is thought to result from a process of altered portal blood flow leading to a portal obliterative venopathy. According to this theory, thrombosis of portal venules occurs secondary to intrahepatic vasculitis due to toxins or autoimmune processes or recurrent microembolization from the portal system or spleen [12]. The resultant endothelial injury and vascular inflammation lead to red blood cell deposition into the space of Disse and luminal narrowing of the small portal vein radicles, contributing to portal hypertension. This area of decreased perfusion leads to local hepatocyte ischemia with resultant compensatory hypertrophy of unaffected adjacent hepatocytes. Ultimately, this process leads to the development of noncirrhotic intrahepatic portal hypertension (NCIPH).

NRH is associated with a broad range of disease processes of hematologic (polycythemia vera, chronic myelogenous leukemia, Hodgkin's disease, and non-Hodgkin's lymphoma), autoimmune (SLE, rheumatoid arthritis, and inflammatory bowel disease), vascular, and infectious origin. An association with chemotherapeutic (thiopurines, oxaliplatin) and antiretroviral therapies (didanosine) has also been described [5–10].

The clinical presentation of NRH is variable and ranges from completely asymptomatic to symptoms of overt portal hypertension. Early and accurate diagnosis of NRH remains a challenge, as most patients are initially asymptomatic, some for prolonged periods of time. While being important to rule out other causes of NCIPH (e.g., steatohepatitis), NRH should be considered in the differential diagnosis of any patient with known risk factors, who presents with signs or symptoms of portal hypertension. The earliest marker of disease is often mild elevations in liver enzymes, which occur in 11–25% of patients [12]. A mild thrombocytopenia associated with NRH has also been described, potentially related to splenic sequestration [8].

As in our case, esophageal varices with or without acute upper gastrointestinal hemorrhage is the most common presenting symptom. Overall, it is estimated up to fifty percent of patients with NRH will develop clinical symptoms [13]; however, it is difficult to define the true prevalence and natural history of NRH as published reports are significantly biased toward symptomatic cases.

Hepatic imaging of NRH demonstrates variable findings, and the differential includes FNH, hepatocellular adenoma, large regenerative nodules, and metastatic disease. Computed tomography can show normal hepatic parenchyma, numerous small nodules, or larger coalesced nodules spanning several centimeters. On nuclear medicine imaging, these lesions may take up sulfur colloid but will remain iso- or hypodense in both arterial and portal venous phases, which can distinguish NRH from FNH [14]. The use of MRI to enhance diagnostic accuracy is still controversial. NRH lesions appear hyperintense on T1-weighted imaging and iso- or hypointense on T2 images; however, the sensitivity and specificity are variable based on recent reports [15, 16].

Liver biopsy remains the only method to definitively diagnose NRH. As evidenced by our case described above, the use of needle and core biopsies can result in inadequate tissue sampling and missed diagnosis. Additionally, coexisting conditions such as fibrosis from hepatitis infection, alcoholic, and nonalcoholic liver diseases can mask the existence of NRH. Reticulin staining of biopsy specimens can highlight areas of focal hepatocyte atrophy and adjacent areas of regeneration without intervening fibrosis, aiding in the histologic diagnosis. Ultimately, the diagnosis of NRH rests on a combination of clinical suspicion, imaging findings, and histologic confirmation of an adequate tissue sample, which in some cases requires wedge biopsy.

There are no prospective or controlled studies in the literature regarding best treatment strategies for complications of NRH. The primary goal is early detection among patients with known risk factors. Once the diagnosis has been established, treatment is aimed at preventing the progression of disease (e.g., cessation of causative medication) or managing the symptoms of portal hypertension. Literature reports of patients with NRH and portal hypertension describe variable success rates with medical management of symptoms. In case of failure, most of the literature reports center on the use of transjugular intrahepatic portosystemic shunt (TIPS) for decompression or liver transplantation. Upon literature review, we could only find one article published in 1991that described surgical shunting for NRH, with no publications in the past 20 years [12].

In our case, an initial treatment approach of beta-blockers, cessation of AZA, and repeated endoscopic band ligation failed. However, due to the absence of parenchymal liver disease, preserved liver function, and no encephalopathy, it was felt that decompression of the portal hypertension would resolve the patient's symptoms without subjecting her to a liver transplant and increased immunosuppression with its inherent side effects. Given the fact that the patient was only thirty-seven years old and had minimal medical comorbidities, surgical shunting was deemed to be more appropriate than TIPS. This was based on the fact that although both surgical shunting and TIPS provide significant portal decompression, TIPS is associated with a higher incidence of variceal rebleeding and dysfunction (e.g., stenosis, thrombosis, and occlusion) with reintervention rates of 48–82% compared with 6.3–11% for surgical shunts [17, 18]. These high reintervention rates offset the initial cost-savings of a TIPS procedure and require close long-term surveillance to monitor for signs of TIPS failure [19].

## 4. Conclusion

While TIPS has become the more common procedure for management of refractory varices, in specific cases of NCIPH with preserved liver function such as NRH, definitive treatment with surgical shunting should be considered in the event that medical management fails. When performed in an experienced center, the procedure carries a low morbidity and offers better long-term durability with lower reintervention rates.
